# Need for Cardiovascular Risk Reduction in Persons With Serious Mental Illness: Design of a Comprehensive Intervention

**DOI:** 10.3389/fpsyt.2018.00786

**Published:** 2019-02-08

**Authors:** Arlene T. Dalcin, Gerald J. Jerome, Lawrence J. Appel, Faith B. Dickerson, Nae-Yuh Wang, Edgar R. Miller, Deborah R. Young, Jeanne B. Charleston, Joseph V. Gennusa, Stacy Goldsholl, Ann Heller, A. Eden Evins, Corinne Cather, Emma E. McGinty, Rosa M. Crum, Gail L. Daumit

**Affiliations:** ^1^Department of Medicine, Johns Hopkins University School of Medicine, Baltimore, MD, United States; ^2^Department of Kinesiology, Towson University, Baltimore, MD, United States; ^3^Sheppard Pratt Health System, Baltimore, MD, United States; ^4^Massachusetts General Hospital, Harvard Medical School, Boston, MA, United States; ^5^Department of Health Policy and Management, Bloomberg School of Public Health, Johns Hopkins University, Baltimore, MD, United States; ^6^Department of Epidemiology, Bloomberg School of Public Health, Johns Hopkins University, Baltimore, MD, United States

**Keywords:** serious mental disorder, cardiovascular risk, intervention, care coordination, behavioral coaching

## Abstract

Persons with serious mental illness (SMI) comprise a high-risk group for cardiovascular disease (CVD)-related mortality with rates at least twice those of the overall US. Potentially modifiable CVD risk behaviors (tobacco smoking, obesity, physical inactivity, unhealthy diet) and risk factors (hypertension, diabetes, dyslipidemia) are all markedly elevated in persons with SMI. Evaluations of programs implementing integrated medical care into specialty mental health settings have not shown meaningful effects on CVD risk factor reduction. Rigorously tested, innovative interventions are needed to address the large burden of CVD risk in populations with SMI. In this article, we describe the design of a comprehensive 18-month intervention to decrease CVD risk that we are studying in a randomized clinical trial in a community mental health organization with psychiatric rehabilitation programs. The individual-level intervention incorporated health behavior coaching and care coordination/care management to address all seven CVD risk behaviors and risk factors, and is delivered by a health coach and nurse. If successful, the intervention could be adopted within current integrated care models and significantly improve the physical health of persons with SMI.

## Introduction

Persons with serious mental illness (SMI) comprise a high-risk group for cardiovascular disease (CVD)-related mortality with rates at least twice those of the overall US (1–5). Potentially modifiable CVD risk behaviors (tobacco smoking, obesity, physical inactivity, unhealthy diet) and risk factors (hypertension, diabetes, dyslipidemia) are all markedly elevated in persons with SMI (6–10). The American Heart Association set strategic goals to improve cardiovascular (CV) health to ideal levels for each risk factor and reduce CVD mortality by 20% for all Americans by 2020 (11). However, persons with SMI often have challenges in everyday functioning due to cognitive impairment and ongoing psychiatric symptoms (12, 13). Together with high prevalence of substance use, unemployment, low-income and social isolation, these factors make implementing effective CVD risk reduction interventions challenging for this population (13–17). Adapted behavioral weight loss interventions and smoking cessation interventions tailored to persons with SMI have shown to be efficacious in clinical trials (18–21), however, evidenced-based interventions are needed that address more than one CVD risk factor. Without special efforts to develop interventions that promote CV health and control risk factors in persons with SMI, health disparities will persist and likely worsen.

While in the general population there is substantial evidence that non-physician interventionists (e.g., care managers) who facilitate and coordinate treatment with primary care physicians (PCPs) can improve multiple CVD risk factors (22–28), evidence for effective interventions in populations with SMI is needed. To address the premature mortality from CVD and other medical causes (3, 29–32) and need for improved access to quality care for physical health conditions (33, 34) among people with SMI, there has been a recent increase in implementation of programs integrating medical care into mental health settings (35–45).

In the US, one such model is the behavioral health home (BHH), which incorporates primary care coordination and management into the specialty mental healthcare setting (35–42). However, results from evaluations of BHHs and other integrated programs to-date have been mixed with either no or minimal effects on actual CVD risk factors (37, 46–51).

Thus, rigorously tested, innovative interventions are needed to address the large burden of CVD risk in populations with SMI. Here we describe the design and implementation of a comprehensive risk reduction intervention incorporating health behavior coaching and care coordination/care management to address all CVD risk factors in persons with SMI that we are studying in a randomized clinical trial.

## Trial Study Design

This community organization-based two-arm randomized clinical trial (the IDEAL trial) tests an 18-month comprehensive, practical risk reduction program compared to control in reducing CVD risk as assessed by the global Framingham Risk score (52) in persons with SMI. Participants were randomized to receive a control condition or the active intervention, which incorporates health behavior coaching and care coordination/care management to improve CVD risk behaviors and factors. Individual CVD risk behaviors and factors comprise secondary outcomes.

## Participants and Settings

Two hundred sixty-nine participants with SMI 18 and older and at least one CVD risk factor (hypertension, diabetes mellitus, dyslipidemia, current tobacco smoker, or BMI ≥ 25 kg/m^2^) were enrolled in the study. Exclusion criteria included having a CVD event in the prior 6 months, a serious medical condition limiting life expectancy, or an active alcohol or substance use disorder, pregnancy, planning on leaving the community in 6 months or the geographic area in 18 months. Serious mental illness diagnosis was assessed by chart review. The study was conducted in partnership with a large community mental health organization that provides outpatient services including psychiatric rehabilitation programs (PRPs). PRPs serve persons with SMI offering vocational and skills training, coordination of behavioral health and social services, and normally provide breakfast and lunch to attendees. We implemented the study at four organization sites with PRPs. As part of our partnership with the community mental health organization and to encourage environments conducive to healthy behaviors, we provided training and resources to promote group physical activity classes open to all program attendees, and a dietician to consult with PRP kitchen staff to provide healthier meals.

## Intervention Overview

We adopted the American Heart Association (AHA) Life's Simple 7 goals and modified some (e.g., 10 lb. weight loss instead of BMI < 25) to provide intermediate targets achievable over the 18-month intervention (11). (Table 1) To work toward these goals, participants in the active intervention met individually with a health coach and a nurse who provided: (1) tailored CVD risk reduction education and counseling; (2) collaboration with physicians to advocate for appropriate management of CVD health risks; and (3) coordination with mental health staff and social supports to encourage and motivate participants to reach individually tailored CV health goals.

**Table 1:** Intervention approaches in comprehensive cardiovascular risk reduction intervention for adults with serious mental illness.

**CVD risk behavior or risk factor targets**	Evidence-based goals^*^	**Intervention approach**
Tobacco smoking	Quit smoking	• Smoking cessation behavioral counseling tailored to readiness to quit • Communication with psychiatrist about pharmacotherapy • Contingency management with CO testing
Diabetes mellitus	Control blood sugar, HbA1c < 7%	• Diabetes self-management education • Behavioral counseling to support medication adherence, healthy diet and exercise • Regular monitoring of blood glucose and communication/coordination with physician, mental health program staff and caregivers
Hypertension	Control blood pressure, systolic blood pressure < 140 mmHg, diastolic blood pressure < 90 mmHg	• Hypertension education • Behavioral counseling to support medication adherence, healthy diet and exercise • Regular blood pressure monitoring and communication with PCPs about monitoring and treatment goals.
Dyslipidemia	Lower cholesterol, total cholesterol < 200 mg/dl, LDL < 130 mg/dl^**^	• Behavioral counseling to support medication adherence, healthy diet and exercise • Communication with the PCP around guideline-concordant treatment with lipid lowering medications.
Overweight/obesity	Healthy weight, encourage 10 pound weight loss for those overweight or obese	• Lifestyle behavioral counseling including simplified dietary messages (e.g., avoid junk food, avoid sugar drinks, limit snacking, limit portion sizes), exercise, weekly weigh-ins, and self-monitoring using paper trackers. • Reinforce the role of weight management in the relevant CVD risk.
Unhealthy diet	Customized based on clinical condition	• Lifestyle behavioral counseling with clear message for healthy diet in context of other risk factors • Self-monitoring to increase awareness and accountability of food choices • Role playing and skill building to support decisions for healthy choices
Physical inactivity	Moderate intensity physical activity, 150 min/week	• Encourage participation in group exercise at PRP • Identify additional exercise strategies e.g., providing pedometers and setting daily step goals, exercise DVDs to guide home-based workouts • Link the role of exercise to the relevant CVD risk.

* *Based on AHA Strategic Goals and prevailing national guidelines modified for the trial, and tailored to participant needs*.

** *CVD risk-based target also used (53)*.

## Theoretical Approach

Behavioral aspects of the IDEAL intervention incorporate social cognitive theory and behavioral self-management concepts (54, 55). Motivational interviewing, is well suited for adults with SMI in that it is patient-centered, and uses a guiding style to enhance intrinsic motivation to improve CVD risk-related behaviors and promote medication adherence (56–59). The intervention also used solution-focused therapy, a positive-oriented, goal-directed technique emphasizing solutions rather than problems (60, 61). These approaches are useful in helping individuals gain confidence from small successes while staying focused on positive long-term changes.

The intervention fits well within a psychiatric rehabilitation framework because rehabilitation models emphasize skill building and goal setting and incorporate reinforcements as part of a behavioral change strategy (62–65). Evidence demonstrates the success of points/reward systems for persons with SMI in improving social interactions, work-related task performance, and other adaptive behaviors (66–74). The IDEAL intervention uses a point system to reward recommended behaviors (e.g., low carbon monoxide (CO) values consistent with no recent smoking, attending group exercise classes). Points can be traded for inexpensive items (e.g., pen, key chain) or saved for larger rewards (e.g., CD player).

The intervention is tailored to meet the cognitive needs of adults with SMI, characterized by memory and executive function deficits (75, 76). Participant materials have high readability and simplicity of messages, and streamlined paper tracking logs correspond to the intervention's lifestyle change approach. In addition, in-person sessions include techniques such as emphasizing learning through repetition, modeling and demonstrating a few specific skills repeatedly; breaking material into small units; and rehearsing behavioral skills (12, 77, 78).

The intervention utilizes concepts drawn from the overlapping constructs of care management and care coordination. Care management is a patient-centered, team-based approach to increase delivery of evidenced-based care for chronic medical conditions (79). Care coordination emphasizes the deliberate organization of patient activities and the sharing of relevant information among all concerned parties to achieve better patient care (80). The IDEAL intervention team includes a nurse and a health coach, who work together to facilitate communication and coordination between participants, providers, and mental health program staff for all aspects of CVD risk factor management.

## Intervention Staff

The health coach is a facilitator with training in health behavior change and individual-level counseling with a skill level typical for a community health educator. When possible, the coach is an employee of the mental health organization; the position is modeled after one that would be feasible and sustainable in a community mental health setting. The coach, embedded in the PRP, is well-positioned to work with (other) community mental health program staff and take advantage of everyday interactions with staff and participants to encourage and support health behavior change and facilitate coordination of care for CVD risk factors. The registered nurse is an intervention team member who serves as a clinical resource addressing all aspects of CVD risk factor status. The coach and nurse report to an intervention director with motivational interviewing and health behavior change expertise. The intervention director implements quality assurance (Table 2), and may also provide direct services to participants.

**Table 2:** Quality assurance techniques used in comprehensive cardiovascular risk reduction intervention for adults with serious mental illness.

**Quality assurance technique**	**Intervention approach**
Training	• Coaches receive initial training on CVD risk behaviors and risk factors • All staff trained on behavior change theories, working with persons with SMI, motivational interviewing, implementing the intervention according to the manual of procedures • Quarterly follow-up training • Follow up training topics determined from trends observed during case management and fidelity observations.
Case Management Meetings	• Used to review each participant's CVD risk factors and current treatment plan and to help facilitate behavior change and/or appropriate monitoring or treatment • Ensure that all participants are reviewed regularly; those with uncontrolled risk factors are prioritized. • Facilitated by intervention director, attended by intervention team and as needed by investigators with expertise in CVD risk reduction strategies
Intervention Fidelity	• Health coach and nurse sessions are either observed in person or audio recorded and reviewed by the intervention director for adherence to the protocol. • Fidelity reviews occur more frequently initially and then at least quarterly to identify skills in need of improvement.

## Intervention Approach

The core elements of the intervention include health behavior coaching, coordination of care and care management.

### Health Behavior Coaching

Participants received 20–30 min individual coaching sessions, with weekly sessions for the first six months and at least biweekly thereafter, based on participant need. Guided by the participant's abilities and interests, the health coach addressed CVD risk and targeted behaviors either simultaneously or sequentially. The sessions used motivational interviewing strategies with an initial focus on building rapport and establishing a working alliance. Sessions included a review of the participant's risk behaviors to identify knowledge deficits and to set short-term behavioral goals. The coach used solution-focused therapy to support attainment of individual goals. Participants were encouraged to self-monitor with simple tracking tools as appropriate for their cognitive abilities. The coach reviewed tracking data with participants to reinforce progress, identified triggers and high-risk situations, and formulated new behavioral plans. The nurse provided educational and counseling sessions related to specific CVD risk factors (e.g., diabetes). The intervention sessions may include family members or peers to support the participant for better control of risk factors (e.g. tobacco smoking).

### Care Management and Care Coordination

The nurse mets with participants for medication-related counseling to address issues with adherence and plan for physician appointments, prioritizing those with uncontrolled risk factors. He/she shared information on participant's CVD risk factor profile with the primary care physician (PCP) and accompanied the participant on selected office visits. The nurse provided information on guidelines for diabetes, hypertension, dyslipidemia and smoking cessation as needed to participants' physicians, and advocated for evidenced-based monitoring and treatment (19, 53, 81–91). If the participant smoked and was interested in pharmacotherapy, the nurse communicated with the psychiatrist or PCP about prescribing smoking cessation medication and supported the participant through the process. The intervention staff coordinated with mental health staff, therapists, caregivers, physicians and/or office staff to support and reinforce participant health goals. This included facilitating the scheduling of and participant attendance at medical appointments, and removing barriers to obtaining medications and supplies as needed.

### Approaches by Risk Factor

Participants received intervention sessions tailored to their individual CVD risk profiles and readiness to address each risk factor. When participants had multiple risk factors, in keeping with a patient-centered approach, intervention staff worked collaboratively with the participant to identify the focus of the sessions. They used options tools, cards with pictures, for possible conversation topics based on the participant's risk factors (e.g., for someone who smokes and has diabetes the options listed would read: Smoking, exercise, medication, sugar drinks). These tools gave the participant the autonomy to choose specific behaviors to discuss, while also allowing the coach to provide direction to the session.

#### Tobacco Smoking Cessation

Intervention staff assessed the motivation of participants who smoke with questions based on the transtheoretical stages of change (92) and placed them in one of three tracks: interested in quitting in 30 days; interested in quitting/reducing smoking but not ready to set a quit date; and expressing no interest in quitting. Those ready to quit within the next month received smoking cessation sessions and those interested but not ready to set a quit date received a motivational enhancement program (19, 93–95). The programs identified personalized reasons to stop smoking, provided training in strategies to respond to urges to smoke (The 4Ds: delay, do something else, deep breathe, drink water), and educated patients about the benefits of quitting and of using smoking-cessation pharmacotherapy. Those in the motivational enhancement program transitioned to quit smoking sessions when ready. These interventions incorporated a breathalyzer that measures expired CO as a marker of recent smoking, with incentives for low CO values. For those willing to consider recommended pharmacotherapy, the nurse worked with participants' physicians to facilitate prescription of varenicline or bupropion, and/or provided dual form nicotine replacement therapy as appropriate. Participants who expressed no interest in quitting smoking focused on other behavior change topics, however, the coach recommended change in smoking behavior monthly and re-assessed willingness to quit. If participants expressed interest in quitting smoking or were ready to set a quit date, they transitioned to another track.

#### Diabetes

Intervention staff helped participants optimally manage their diabetes through self-management education sessions, supporting medication adherence, and encouraging self-monitoring of blood sugar as appropriate. They facilitated hemoglobin A1c monitoring and other guideline concordant care for diabetes with the PCP. For those with uncontrolled diabetes, the nurse and coach coordinated care as needed to decrease barriers to blood glucose control (e.g., obtaining and using glucometer) with mental health staff, caregivers, PCPs and/or specialists. They encouraged high impact dietary behaviors (e.g., no sugar drinks), participation in regular exercise, and weight loss when appropriate.

#### Hypertension

The nurse met with participants for hypertension education sessions including discussion of medication adherence. Intervention staff monitored blood pressure regularly and communicated with participants and PCPs about monitoring and treatment goals. The nurse also reinforced the dietary (e.g., avoid salty/processed foods) weight loss and exercise goals set with the coach.

#### Dyslipidemia

The nurse met with participants who met recommended guidelines for lipid lowering therapy for medication education and counseling before communicating with the PCP for guideline-concordant treatment. Both the coach and nurse encouraged high impact dietary behaviors (e.g., no greasy foods), participation in regular exercise, and weight loss if needed.

#### Overweight/Obesity

The coach used lifestyle strategies that have proven effective in previous clinical trials including individual counseling sessions, weekly weigh-ins, simplified dietary messages (e.g., avoid junk food, avoid sugar drinks, limit snacking, limit portion sizes) and paper trackers to help monitor these behaviors (18). The nurse also linked the roles of weight management to the participant's other CVD risks.

#### Unhealthy Diet

Intervention staff provided guidance on ways to eliminate unhealthy dietary choices and incorporate healthier options in the context of other CVD risk factors and the participant's health goals. The conversations used simplified messages and incorporate paper trackers to facilitate awareness and goal setting. Dietary topics included: Avoiding salty/greasy foods, sugar drinks, sweets and processed foods, and eating smart portions and more fruits and vegetables. The coach used role playing and hands-on activities (e.g., visiting the soda machine) to model decision making for healthy diet choices.

#### Physical Inactivity

The intervention staff encouraged participation in regular moderate-intensity physical activity. We trained PRP staff to deliver site-wide group exercise classes using either a study-made DVD or commercially available walking DVD and recommend that classes are offered three times per week. The health coach encouraged exercise class attendance for intervention participants, participated in some of the classes, and substituted for PRP staff if requested. The coach worked with intervention participants to identify additional strategies to meet physical activity recommendations, for example, providing pedometers and setting daily step goals, and providing exercise DVDs to guide home-based workouts. The nurse reinforced exercise goals set with the coach and linked the importance of exercise as an independent health behavior and to the relevant CVD risks.

## Discussion

Given the extraordinarily high and persistent burden of CVD risk factors and resultant CVD in persons with SMI, there is an urgent need for tailored, effective interventions to promote CV health. We describe a comprehensive health behavior coaching and care coordination/care management intervention designed to address each CVD risk behavior and risk factor for adults with SMI in community mental health settings. The IDEAL intervention design has important strengths. First, the intervention uniquely focuses on addressing all of the seven CVD risk factors and behaviors with strategies directed at reaching clinically significant targets consistent with treatment guidelines. Second, the health coach is embedded in the PRP, facilitating coordination and communication with mental health staff in the everyday community mental health outpatient environment. This provides a practical real-world design to optimize future implementation feasibility and sustainability.

The IDEAL intervention faces some challenges to success. The intervention is ambitious in its attempt to address multiple risk factors in persons with SMI given that accomplishing reduction for a single risk factor in those without SMI often takes intensive efforts (96). Fortunately, the intervention's lifestyle-based recommendations to help address risk factors such as high blood pressure or high blood glucose involve similar approaches. Having both a coach and nurse use a coordinated approach for each participant should help to address this challenge. Another potential barrier to success is that the intervention team does not prescribe medications, and PCPs in the community have no specific incentive to participate in the intervention's care coordination efforts.

If shown to be effective in the RCT, the IDEAL intervention could be implemented through programs, like the BHH, which provide a financing mechanism for staff (e.g., nurse) and infrastructure to integrate care coordination and management into specialty mental health settings. BHHs are currently operating in 15 U.S. states and D.C. through the Affordable Care Act's Medicaid health home waiver program, and have been implemented through the Substance Abuse and Mental Health Services Administration's Primary Behavioral Health Care Integration initiative and other local programs (35, 36, 38).

However, BHHs generally do not require use of a specific care coordination/management protocol or intervention, thus the strategies currently employed in BHH models vary considerably across sites; they also typically do not emphasize evidenced-based behavioral counseling for CVD risk behaviors (35–44). These features likely contribute to the mixed results to-date for BHHs. The two US RCTs evaluating BHHs found the programs improved quality of primary care delivery; while one showed small reductions in cardiovascular risk among a subset of participants, the second showed no effects on CVD health outcomes (37, 48). These findings suggest that care coordination alone, while potentially capable of improving processes of care, may not be enough to achieve improved clinical outcomes. In Denmark, the CHANGE trial tested care coordination vs. care coordination plus lifestyle coaching vs. control and found no change in CVD risk or individual risk factors over 2 years (97). These null results may be due to the high quality of the health system in Denmark such that many participants at baseline had controlled risk factors; also, the lifestyle coaching may not have been as intense as needed to affect additional CVD risk reduction.

The IDEAL intervention may be well suited for integration into and could improve the effectiveness of models like the BHH. When embedded into a BHH-like structure with its financing, the intervention with its standards of procedure for all aspects of care coordination and tailored, evidenced-based behavioral counseling, could make considerable progress in improving the CVD health for a population that is not routinely receiving quality CV care.

## Conclusion

Unless effective interventions that effect CV health outcomes are implemented, populations with SMI will continue to lag far behind the nation's CVD goals. Interventions and models incorporating CVD risk factor treatment in mental health settings to-date have not shown meaningful changes in CV risk factors. The IDEAL intervention incorporates care coordination and care management concepts with intensive health behavior change coaching to address all CVD risks in persons with SMI in community mental health outpatient program settings. If successful, the intervention could be adopted within current integrated care models and significantly improve the physical health of persons with SMI.

## Ethics Statement

This study was carried out in accordance with the recommendations of the Johns Hopkins Medicine IRB with written informed consent from all subjects. All subjects gave written informed consent in accordance with the Declaration of Helsinki. The protocol was approved by The Johns Hopkins Medicine IRB.

## Author Contributions

AD, GD, and GJ wrote the manuscript. GD, LA, GJ, FD, N-YW, DY, JC, RC, AD, SG, JG, CC, EE, and AH contributed to the conception, design and implementation of the study. EM wrote a section of the manuscript. All authors contributed to manuscript revision, read and approved the submitted version.

### Conflict of Interest Statement

The authors declare that the research was conducted in the absence of any commercial or financial relationships that could be construed as a potential conflict of interest.
